# Barriers and strategies needed to improve maternal health services among pregnant adolescents in Uganda: a qualitative study

**DOI:** 10.1080/16549716.2022.2067397

**Published:** 2022-06-28

**Authors:** Samuel Nambile Cumber, Catherine Atuhaire, Vivian Namuli, Malin Bogren, Helen Elden

**Affiliations:** a Institute of Health and Care Sciences; The Sahlgrenska Academy at University of Gothenburg, Gothenburg, Sweden; b Faculty of Health Sciences, University of the Free State, Bloemfontein, South Africa; c Section for Epidemiology and Social Medicine; Department of Public Health, Institute of Medicine (EPSO), The Sahlgrenska Academy at University of Gothenburg, Gothenburg, Sweden; d Faculty of Medicine, Department of Nursing, Mbarara University of Science and Technology, Mbarara, Uganda; e Region Västra Götaland, Department of obstetrics and gynaecology, Sahlgrenska University hospital, Gothenburg, Sweden

**Keywords:** Adolescents, experiences, maternal health services, pregnancy

## Abstract

**Background:**

In Uganda, the uptake of maternal health services is very low, with only 41.1% of pregnant adolescent girls attending the eight antenatal visits that are recommended by the World Health Organisation. Uptake of maternal health services is essential in reducing the current level of adolescent pregnancies as well as its adverse effects on adolescent mothers and their babies, such as preterm deliveries, prolonged labour, death during pregnancy, and childbirth. No previous study has described pregnant adolescents’ experiences with maternal health services in Uganda.

**Objective:**

This study aimed to describe the barriers and strategies needed to improve maternal health services among pregnant adolescents in Uganda.

**Methods:**

Data were collected in the Naguru Teenage Information and Health Centre in Uganda through individual interviews involving 31 pregnant adolescents. The transcribed interviews were inductively analysed through content analysis.

**Results:**

The pregnant adolescents described difficulty in reaching, as well as lack of financial support to visit, the Naguru Teenage Information and Health Centre, which is a clinic providing youth friendly services. Feelings of being discriminated against and disrespected by health workers, and lack of privacy when receiving health services was major barriers that hindered their access to maternal health services. Pregnant adolescents’ access to these services can be enhanced by improving health workers’ working conditions, accelerating community and health worker awareness on ways to mitigate these barriers, and developing policies that encourage men’s involvement in maternal health services.

**Conclusion:**

Adolescents in Uganda face considerable barriers to accessing improved and quality maternal health services. To mitigate these barriers, according to the adolescents, considerable efforts are required to tackle health workers’ working conditions and sensitise the community on the importance of, as well as securing the availability of, maternal health services for pregnant adolescents. Future research should focus on pregnant adolescents who receive family support.

## Background

Accessing maternal health care remains a global challenge, with over 75% of births worldwide reported to occur outside formal health facilities [1]. In Uganda, the situation is similar to that in other resource-constrained countries, as only 65% of childbirth deliveries are performed by skilled health workers [1]. Achieving the Sustainable Development Goals (SDGs), especially the third goal on health, which aims to reduce the global maternal mortality ratio to less than 70 per 100,000 live births by 2030, will require improved access to quality maternal services among the most vulnerable groups of women [2].

Adolescents constitute one of the most vulnerable groups as they are underserved by the current maternal health service delivery in Uganda and other low-income countries [1]. Also, adolescence is a critical period, involving the transition from childhood to adulthood, which is characterised by dramatic physical, sexual, psychological, and social changes [3]. However, the current investments in maternal health for adolescent girls do not match the existing needs, which produces dire consequences for pregnant adolescents and their un-born babies [2]. Complications during pregnancy and childbirth are among the leading causes of death among adolescent girls worldwide [2]. Thus, adolescent pregnancy is one of the adverse consequences of unmet maternal health needs [3,4]. The World Health Organisation (WHO) reported that, globally, over 20% of adolescent girls give birth before the age of 18, and over 16 million adolescent girls aged 15–19 years give birth annually [5]. Adolescent pregnancy is more prevalent in Sub Saharan Africa, where one-third of the adolescent girls give birth before the age of 18 [3].

Uptake of maternal health services is essential in reducing the current level of adolescent pregnancies and its adverse effects on adolescent mothers and their babies, such as preterm deliveries, prolonged labour, and death during pregnancy and childbirth [4–6]. Promoting maternal health services among adolescents is aimed at reducing the rate of adolescent pregnancies and simultaneously support adolescent girls to have safe and healthy pregnancy and childbirth experiences and deliver healthy new born babies by increasing the access and utilisation of professional maternal health care [5]. WHO recommends a continuum of maternal health services, ranging from antenatal care, birth, and postnatal care, owing to the existing evidence that access to a set of these services greatly improves pregnancy outcomes [3].

The literature on factors that influence the low uptake of maternal health services among pregnant adolescents has advanced many factors such as uncoordinated policies, knowledge and attitudes of adolescents and parents, poor health service delivery, and poverty [7,8].

In Uganda, where adolescents constitute 34.8% of the total population, the Ministry of Health prioritises adolescents’ health by improving access to contraceptives, professional care during childbirth, and postnatal care; however, uptake remains low [9]. The 2020 Uganda Demographic and Health Survey [10] reported that 24.8% of girls aged 15–19 had begun bearing children. Over 30.4% of 15–19-year-old girls who require contraceptives are unable to access such services, while 17.2% of adolescent girls die because of childbirth-related complications [10]. In addition, only 41.1% of pregnant adolescent girls attended the eight antenatal visits that are recommended by the WHO [11].

In Uganda, youth-friendly services remain scarce, with only 10% of the health facilities in the country offering such services [12]. To our knowledge, no earlier study has described pregnant adolescents’ experiences with maternal health services in Uganda. Accordingly, this study aimed to describe the barriers and strategies needed to improve maternal health services among pregnant adolescents in Uganda.

## Methods

### Study design and settings

The study used a qualitative design to describe pregnant adolescents’ experiences with maternal health services in Uganda. A qualitative method is used when there is limited knowledge on a study topic [13].

The study was performed at the Naguru Teenage Information and Health Centre (NTIHC). The NTIHC is among the few clinics that provide youth-friendly services. It operates at the Kiswa Health Centre and provides support to seven health centres in Kampala and 24 public health centers in eight neighbouring districts. The NTIHC provides sexual and reproductive health services, including counselling and care during pregnancy as well as postnatal care, family planning, prevention, and treatment of sexual transmitted diseases (STIs) and human immunodeficiency virus (HIV), youth counselling, and prevention and control of alcohol and drug abuse. All care is free of charge, targeting young people aged 10–24 years, most of whom are from slum areas.

### Participants and data collection

The participants included 31 pregnant adolescent girls, aged 15–19 years, mean 17 years, seeking maternal health services at the NTIHC from February 1 to 28, 2019. They lived either alone or with their adolescent spouses or friends, rather than with their parents or legal guardians. They have diverse economic backgrounds: 5 employed and 26 unemployed girls. The education level for 21 participants was senior two, while 10 had dropped out of school at the primary level. For details about the adolescent’s characteristics, see Table 1.

**Table 1.** Characteristics of the 31 participants included in the study. Values are numbers.

Variables	n
**Age (years)** 15 16 17 18 19	2 4 9 11 5
**Gestational weeks at time of interview** 10–20 21-25 26–30 31-35	4 1 10 16
**Level of education** No education Primary school Secondary school Tertiary level/College	8 10 11 3
**Employment status** Employed Unemployed	5 26
**Antenatal visits, n** 1 2 3 4 5 6 7	14 4 6 2 2 2 1

Participants in this study were selected through a convenient sampling technique. Two of the authors (CA and VN) contacted the managers at the centre and informed them about the study. The managers gave them access to the health facility registry to check out the names, phone numbers and addresses of the adolescent pregnant girls that had visited the health centre during the data collection period. One of us authors called or visited 49 pregnant adolescents and explained the aim of the study. Twenty-four pregnant adolescent girls decided to participate in the study during the phone call and seven agreed to participate in the study during our visit in their homes. Appointments were made for the interviews and the participants were given the opportunity to choose a convenient place and time for the interview. However, most of them preferred to have the interviews at the health centre during their re-visit for antenatal check-up though some preferred to have the interviews in their homes and some at their places of work. The participants gave their written consent before the interview. Data were collected using face-to-face interviews. The interviews were conducted by one of the authors (VN) in either English and/or the native language (Luganda). The interviewer created an open inclusive climate for the adolescents to express their experiences when assessing maternal health services. The interviewer made sure that all interviews were conducted in a quiet place to avoid disruptions and ensure privacy and confidentiality. The interview started with an opened-ended question: Please tell me about your experiences of the maternal health services? It was followed by question in an interview guide (see Appendix A). Follow up questions as ‘can you tell me more’ and ‘can you give more examples’ were also used. The questions sought to explore the pregnant adolescents’ experiences with maternal health services in Uganda. Each interview lasted for 40–50 minutes and was audio-recorded. If negative experiences were revealed in the interviews, a follow-up meeting was offered. The interview guide, workbooks, and other study materials were stored safely in a locker in a safe location at the university and secured with a lock.

### Data analysis

The interviews were transcribed verbatim from Luganda to English and the data were manually analysed. Data analyses were conducted using an inductive content analysis [14], which is the process of analysing data by identifying, coding, and categorising the primary patterns that emerge from the collected data. First, the authors read the transcripts several times to familiarize themselves with the data. Next, in new readings, meaning units were marked, compared, and sorted into codes, which were then compared and clustered into subcategories and generic categories. After several analysis refinements, from text details to wholeness, the results were finalized [14]. Table 2 presents the data analysis process.

**Table 2.** Illustration of the data analysis process.

Meaning units	Codes	Sub Categories	Generic Categories
I am financially handicapped, and I cannot afford some services. They are surely expensive for me	Lack finances	Lacking financial support	Barriers to Accessing maternal health services
The number of health workers should be increased	Increase number of health workers	Working conditions for health workers need to improve	Strategies needed for adequate maternal health services

## Results

Two generic categories: ‘barriers to accessing maternal health services among pregnant adolescents’ and ‘strategies needed for adequate maternal health services’ for improving access to maternal health service emerged from the analysis. For an overview of the Generic Categories and Sub Categories, see Table 3. Selected quotes from the interviews were included so that the readers may evaluate both the interpretations and credibility of the analysis. Quotations from the 31 participants are labelled PA 1–31.

**Table 3.** Generic categories and subcategories describing pregnant adolescent’s experiences with maternal health services.

Generic Categories	Sub Categories
Barriers to accessing maternal health services	Lacking financial support
	Difficulties in reaching health facilities
	Experiencing discrimination, disrespect, and lack of privacy
Strategies are needed for adequate maternal health services	Working conditions for health workers need to improve
	Awareness in community and health workers needs to be accelerated
	Men should be involved in maternal health care service

### Barriers to accessing maternal health services

Pregnant adolescents revealed varying experiences regarding the barriers to accessing maternal health care services in Uganda. These barriers were identified as lacking financial support, difficulties in reaching health facilities, and experiencing lack of privacy, discrimination, and disrespect by health workers when receiving health services.

#### Lacking financial support

As identified by adolescents, lacking financial support during pregnancy was a barrier to accessing maternal health services. This lack was mainly explained in terms of the failure to cater for transport, acquire prescribed drugs, and maintain a balanced diet, as recommended by health workers.

*I am financially handicapped, and I cannot afford services such as scan fees or drugs. They are surely expensive for me*. (PA 20, 20 years old).

*… the costs are high and unaffordable, while the distance to the health facilities is also terrible because for every antenatal visit, I need to have over USh 20,000 for transport, which I can’t afford* (PA 19, 15 years old).

The pregnant adolescents either were dependents or had businesses that generated very little income, which made it difficult for them to afford transport. Another consequence of not having enough money was the challenge in maintaining a healthy diet or buying recommended drugs. Sometimes, they were asked to pay for health care services, even though these were free of charge. One pregnant adolescent said:

*It is very annoying when, in some health centres, service providers tell us to pay money, yet community leaders tell us that the services are free of charge because the government pays for them*. (PA 5, 17 years old)

#### Difficulties in reaching health facilities

Another barrier affecting pregnant adolescents’ access to maternal health services was the long distances to health facilities. Some of the participants had to travel over two hours to reach the facilities, which was considered challenging, especially when pregnant. Besides the long distance, heavy road traffic caused them to arrive late for their antenatal visits.

*… Besides, the distance from the respective locations to this health facility is another challenge. I come from very far, and it takes me two hours to reach here; and with my health status, travelling for 2 hours to and from the health centres make it hard for me to respond to my antenatal appointments*. (PA 11, 17 years old)

*One needs to be determined and perhaps very sick to travel. … if I have no health problem, I cannot just visit doctor because it’s my appointment* (PA 29, 15 years old).

#### Experiencing discrimination, disrespect, and lack of privacy

Sometimes, they felt condemned, discriminated, and perceived as outcasts when visiting health services. One adolescent said, ‘*Our parents are so disappointed in us, so we can only look up to members in the community for some support’* (PA 8, 17 years old). However, the adolescents expressed that people in their communities did not want to socialise with them either. They also revealed that community members are usually rude to them.

*This makes us feel as if we aren’t supposed to live; for sure, these people can turn you to nothing. This is a terrible scenario*. (PA 15, 18 years old)

Another critical barrier was the attitude of health workers toward pregnant adolescents at the antenatal unit. One pregnant adolescent said: ‘*They do not even show care and concern. They keep blaming us for being stubborn and getting pregnant’* (PA 4, 18 years old). They were not friendly and were disrespectful, nonchalant, and rude to the adolescents. This made the adolescents feel embarrassed.

*They always speak to us rudely and loudly, drawing everyone’s attention on you*. (PA 19, 18 years old).

Other pregnant adolescents pointed out the lack of attention or care from health workers.

*Sometimes you fail to receive medical attention, and the health provider claims that you are late and asks you to return next time. I feel better staying at home than coming to the facility and not being attended to* (PA 21, 16 years old).

Lack of privacy was also a significant barrier to accessing health care at the antenatal unit. One pregnant adolescent described her visit at a registration desk in open space.

*During my first antenatal visit, when I went to the registration desk, I was asked sensitive questions, and the answers I gave drew everyone’s attention to me, and I felt ashamed* (PA 4, 19 years old).

### Strategies needed for adequate maternal health care services

Pregnant adolescents provided a range of possible strategies for improving maternal health services among pregnant adolescents in Uganda. The emergent subcategories are presented below.

#### Working conditions for health workers need to improve

The need for the government to improve health facilities and supply the necessary equipment was highlighted as critical for providing maternal health care services. Pregnant adolescents revealed the need to construct or improve the existing maternal wards and labour suites, and ensure that essential supplies and equipment, such as scan machines and fetoscopes, are available in health care facilities. Increasing the number of health workers and reducing workloads are also important measures for improving health care. Suggestions for improvement are as follows:

*Construct a labour suite for pregnant women, build more health facilities, and install scan machines for pregnant women, and the government should increase the number of health facilities and equip them with drugs* (PA 18, 16 years old).

*The number of health workers should also be increased such that the waiting time is minimised, as this will encourage more adolescents to seek maternal health services* (PA 12, 17 years old).

According to the results, improving the remuneration of health workers is an important strategy for increasing access to maternal health services among pregnant adolescents. They believed that poor remuneration was the cause of high rates of absenteeism and turnover in health facilities. One pregnant adolescent stated that the *government should improve remuneration for health workers so that they are motivated to work* (PA 5, 18 years old).

Poor remuneration was found to result in an inadequate number of health workers, compared to the number of pregnant women seeking their services. This, in turn, results in a long waiting time, which discourages many pregnant adolescents from accessing and utilising maternal health care services.

*Increasing health workers remuneration will reduce the problem of pregnant adolescents and other mothers waiting long at health facilities to see health workers. Waiting for long discourages mothers from coming for more visits* (PA 5, 17 years old).

#### Awareness in community and health workers needs to be accelerated

The importance of sensitising the community and health workers to improve their image of adolescent pregnancy was stressed.

*There is a need to sensitise the community on how to socialise with pregnant adolescents so that they exercise empathy and learn to live with some of us because we need their support too* (PA 3, 15 years old).

*Community members need to improve their attitudes toward adolescents who conceive and need reassurance because we are still young and do not know what we are supposed to do* (PA 8, 17 years old).

#### Men should be involved in maternal health care service

Some adolescents suggested that the government should develop policies that mandate men to accompany their wives while seeking maternal health care services. They mentioned that the lack of male participation in seeking these services affects their access to, and utilisation of, health care services. In addition, pregnant adolescents need support from their spouses to seek maternal services.

*We look up to our men; for that reason, they make most of the decisions about pregnancy. They pay hospital bills and transportation, among other basic needs. It will be good if they accompany us to the hospital, so they can understand the challenges we face on the way and in the hospital’* (PA 8, 17 years old).

## Discussion

Based on their experiences, the pregnant adolescents described the following barriers to accessing maternal health care services in Uganda: lack of financial support, difficulties in reaching health facilities, and experiences with lack of privacy, discrimination, and disrespectful behaviour by the health workers. The strategies required for adequate maternal health care services in Uganda include improving working conditions for health workers, increasing the awareness of the community and health workers, and greater involvement of men in maternal health care services.

### Barriers to accessing maternal health services among pregnant adolescents in Uganda

Most adolescents in this study lacked the financial support needed to access or utilise maternal health services, such as transport costs, prescribed drugs, and a balanced diet, as recommended by health workers. Similar findings on lack of financial support and affordability challenges as barriers to utilisation of maternal health services have been reported by other researchers in contexts similar to Uganda. A scoping study on maternal health service utilisation of adolescent women in Sub-Saharan Africa reported that adolescents who are economically constrained lack resources to spend on health care, including maternal health services. In addition, they are more likely to disengage from social networks, increasing the risk of not being reached by existing programs intended to increase access to maternal health services among adolescents [3]. This argument has been supported by other studies in Africa [15–17].

Adolescents’ difficulty in reaching health facilities was identified as another barrier. The study showed that long distances to health facilities or other maternal health service centres was a serious deterrent for pregnant adolescents. This finding is consistent with those of other studies conducted in Africa. Two studies conducted in Nigeria and Zambia reported a strong correlation between distance to a health facility and the level of adolescents’ utilisation of antenatal care services [14,18]. In similar findings reported by Malawi, longer distance was associated with lower health care utilisation, showing that one additional kilometre resulted in a 1.2% increase in homebirths, 0.8% decrease in seeking at least three antenatal care visits, and 0.8% decrease in skilled attendance during childbirth [19]. Accordingly, a number of studies have recommended increasing the coverage of maternal health services as a key intervention in the search for increased utilisation of maternal services among adolescents [14,20]. Further, the discrimination, disrespect, and lack of privacy experienced by adolescents while receiving health care services was reported as another barrier. Young adolescents face attitudinal barriers and are sometimes stigmatised by health workers, preventing them from fully utilising maternal health services. A study carried out in Nigeria reported that poor attitudes and unprofessional conduct among health workers were responsible for over 25% of the barriers affecting the utilisation of maternal health services [18]. Four other studies qualified this finding, arguing that previous experience with health workers strongly influences future maternal health care seeking behaviour [14,21,22]. Low levels of maternal and paternal education and unmarried mothers are some factors that can lead to discrimination and disrespect in rural settings, and these are significant factors that have been associated with adolescent pregnancy in a study conducted in Sri Lanka [23].

### Strategies for improving adequate maternal health services in Uganda

Improving the working conditions of health workers was found to be a key strategy for improving maternal health services among adolescents in Uganda. Further, staffing, supplies, and equipment within health facilities as well as health workers’ remuneration should be increased. This recommendation is in line with the findings in a study carried out in Nigeria that provision of adequate drugs, recruitment of more skilled health workers, and better remuneration have the potential to increase the coverage of antenatal care by 9.6% [24]. A systematic review of barriers to accessing maternal health services in Africa suggests that health professionals should be empowered with the appropriate knowledge, ethical and medical training, and improved remuneration as a catalyst to providing equitable and high-quality maternal health services. This strategy has also been echoed by two other studies in the African context [25].

The second strategy is to accelerate awareness regarding maternal health services for adolescents among the health workers and the community at large. The current study revealed that increasing awareness of the existing health services is vital in enhancing accessibility to maternal health services. This strategy is supported by the findings of other studies from Nigeria [26] that community involvement through sensitisation enables maternal centres to innovate more resilient and accessible health systems. Likewise, Lassi et al. [27] found that involving communities in reproductive health services also bridges the sensitisation and awareness gap between a community and health services, resulting in improved access to, and improved uptake of, maternal health care services.

The third strategy is to increase men’s involvement in maternal health services because they are responsible for major decisions regarding allocation of resources to various family priorities, including health care. Therefore, involving men in maternal health services increases the chances of increased allocation of resources to maternal health at the family level, including seeking antenatal care services, contraceptives, and childbirths within health facilities [28]. Research shows that involving men in maternal health services can decrease the risks of pregnancy and delivery complications, if they are aware of when they must take the women to a health care facility. As a result, various studies have found that male involvement increases access to antenatal care services, delivery by skilled attendants, utilization of modern family planning methods, and reduction of gender-related barriers to accessing maternal health services [3,29,30].

### Methodological considerations

This study had a few limitations. The results were interpreted based on the context. Qualitative findings being contextual does not mean they have no meaning in other contexts; however, the context changes when findings are transferred [31]. Another limitation is that the participants were only those who attended the youth friendly clinic i.e. NTIHC which could have led to selection bias. One strength of the study is that some participants had completed 26 gestational weeks, and 16 had completed 31 gestational weeks. Most of them had only made 2–3 visits to the hospital. No participant had made eight visits, which is the recommended number of visits. This might have affected the results of the study, either directly or indirectly.

Three of the authors (SNC, CA, and VN) have vast experience in pre-understanding adolescent challenges during pregnancy in the Ugandan setting. This may have a negative influence on the study when gathering data and conducting qualitative research. However, the researchers were aware of this early on; therefore, two co-authors, both midwives, senior researchers, and experts in the field (HE and MB), who did not have experience in the Ugandan context, conducted constant verification and counterbalanced this pre-understanding, enabling the exploration and discussion of the data in a balanced manner.

## Conclusion

No previous study has described pregnant adolescents’ experiences with maternal health services in Uganda, which affirms that access to maternal health services among pregnant adolescents is still a big challenge in the country. However, the results contribute to the existing knowledge on adolescent experiences in accessing maternal health services in Uganda. There are three major barriers to this challenge: lack of financial support for pregnant adolescents to afford maternal health services; difficulties in reaching health facilities; and discrimination, lack of privacy, and disrespect of pregnant adolescents by health workers when they seek health care services. Therefore, the study avers that considerable efforts are required to tackle the barriers that limit access to maternal health services by tackling the working conditions of health workers, sensitising the community on the importance and availability of maternal health services, and increasing the involvement of men. Together, these barriers serve as major risks to providing maternal health services for pregnant adolescent, and this hinder the progress across the SDGs especially regarding health. Based on the lessons learned from this study, we recommend that the government, community, and health workers in Uganda and other settings with similar conditions must play significant roles if maternal health service utilisation is to be enhanced among adolescents. Future research should focus on pregnant adolescents receiving family support.

## Data Availability

The data set used and/or analysed in this study are available from the first author, on reasonable request.

## Paper context

In Uganda, the uptake of maternal health services among pregnant adolescent girls is low. This study shows that difficulties in reaching the health facilities, lack of financial support, feelings of being discriminated and disrespected by the health workers and lack of privacy are barriers. Considerable efforts are required to tackle health workers’ working conditions and sensitise the community on the importance of, as well as securing the availability of, maternal health services for pregnant adolescents.

## Appendix A

In-Depth Interview Guide: Access to maternal health services for pregnant adolescents in Uganda

Date of Interview |___ ___|___ ___|___ ___ ___ ___| [DD/MM/YYYY]

**Socio demographics**

*Questions asked and recorded for each IDI participant*

(I) Age, completed years: |___ ___|

(2) Marital Status: Married |___| Never Married|___| Separated/Divorced|___| Widow|___|

(3) Highest level of education: None |___| Primary|___| Secondary |___| Tertiary |___| University |___|

(4) Occupation: Unemployed |___| Agriculture |___| Unskilled/Manual |___|

Skilled/Manual |___| Trading/Vending |___| Clerical|___|

Professional/Technical/Managerial |___| Student|___|

(5) Household, assets ownership: Land |___| House|___| Car |___|Cattle |___|

Motorcycle |___| Bicycle |___| Computer |___| Refrigerator |___| Microwave |___|

Television |___| Radio |___|Electricity |___| Shop|___|

(6) Parity: One |___| Two |___| Three |___| Four and more |___|

(7) How old is your current pregnancy (gestational weeks)? |___ ___|

(8) How many times have you sought antenatal care|___ ___|

**Questions**:

1. Please tell me about your experiences of the maternal health services.
2. Tell me how you heard about the different maternal health services you mentioned
3. What have you heard about maternal health services?
4. Which maternal health services have you used from any healthcare centre?

b. What maternal service have you mainly used? Has it helped you in any way?

1. Tell me where pregnant adolescent girls in this community can obtain the different services you mentioned
2. I’ve heard some people say that it is no longer important for pregnant mothers to visit a public or private health facilities; what do you think or what is your take on this?
3. How do you feel about the quality of the current maternal health services available at the health facility?
4. How do you feel about the way health workers provide you with maternal health services?
5. Tell me what you think hinders adolescent pregnant girls of this community from accessing maternal health services.
6. What are the challenges you have encountered while accessing maternal health services?
7. What cultural beliefs, norms and values do you know about in your area that discourage you from accessing maternal health services?
8. What personal barriers do you encounter when accessing maternal health care services?
9. What are the suggestions you can make that will help to improve services in the future?
10. How best can maternal health services be rendered to improve accessibility in your opinion?
11. Tell me what motivated you to come here to seek for maternal health services.

Is there anything else that you would like to tell me about the issues we have discussed so far?
